# Development of Rabbit Meat Products Fortified With n-3 Polyunsaturated Fatty Acids 

**DOI:** 10.3390/nu1020111

**Published:** 2009-10-20

**Authors:** Massimiliano Petracci, Maurizio Bianchi, Claudio Cavani

**Affiliations:** Department of Food Science, University of Bologna, 47023 Cesena (FC), Italy; Email: maurizio.bianchi@unibo.it (M.B.); claudio.cavani@unibo.it (C.C.)

**Keywords:** rabbit, feeding, linseed, meat, n-3 PUFA, α-linolenic acid

## Abstract

Rabbit meat is a highly digestible, tasty, low-calorie food, often recommended by nutritionists over other meats. Currently research in the rabbit sector is interested in developing feeding strategies aiming to further increase the nutritional value of rabbit meat as a “functional food” by including n-3 polyunsaturated fatty acids (n-3 PUFA), conjugated linoleic acid (CLA), vitamins and antioxidants in rabbit diets and assessing their effects on both raw and stored/processed meat quality properties. Our recent studies indicate that the dietary inclusion from 3 to 6% of linseed might be considered as a way to achieve the enrichment of the meat with α-linolenic acid and to guarantee satisfactory product stability during further processing and storage. Considering that 6% dietary linseed corresponds to a n-3 PUFA content of 8.5% of the total fatty acids and a lipid content of 4.7 g/100 g of leg meat, a content of 396 mg n-3 PUFA/100g meat can be estimated, which represents about 19% of the recommended daily allowance (RDA) for n-3 PUFA.

## 1. Introduction

European rabbit meat production is approximately 500 thousand tons, corresponding to a 30% share of the world production. It is concentrated in the Mediterranean Region. Italy is by far Europe’s leading producer of rabbit meat, with Spain ranking second, and France third [1]. Rabbit meat is still considered a niche product, especially because of its time consuming preparation which requires culinary skills and because of cultural differences among European consumers. However rabbit meat is often recommended by nutritionists over other meats because it fits well with the current consumer demand for a low-fat meat with a high degree of fatty acid unsaturation and low sodium and cholesterol levels. Moreover, on last years, consumer lifestyle changes in developed countries have led to a meat market more and more focused on easy-handled and processed products (“convenience foods”).

Currently research in the rabbit sector is interested in developing feeding strategies aimed at further increasing the nutritional value of rabbit meat as a “functional food” by the inclusion in rabbit diets of n-3 polyunsaturated fatty acids (PUFA), conjugated linoleic acid (CLA), vitamins and antioxidants and assessing their effects on both raw and stored/processed meat quality properties [2,3]. Despite the limited capacity of metabolic conversion of α-linolenic acid (ALA; C18:3 n-3) to longer chain PUFA such as eicosapentaenoic (EPA; 20:5 n-5) and docosapentaenoic (DHA; 22:6 n-3) acids [4], there are many potential roles in human health for ALA that could be independent from its conversion to DHA [5]. Linseed (or flaxseed) is particularly rich in ALA (50-60% of total fatty acids) and is commonly used as a dietary supplement in humans. Alpha-linolenic acid (C18:3 n-3) is an essential fatty acid in the human diet and the major dietary source of α-linolenic acid are some vegetable oils such as rapeseed and soybean, where it accounts for up to 10% of total fatty acids [6].

The intramuscular fatty acid composition of monogastric animals is a reflection of their dietary fatty acids, while in ruminants biohydrogenation in the rumen (i.e., saturation of the dietary unsaturated fatty acids) is responsible for the smaller variations in intramuscular fatty acid composition [7]. As a consequence, the dietary use of linseed in monogastric animals has been proposed by many authors as a plant-based way to raise the content of n-3 PUFA, and mainly α-linolenic acid (C18:3 n-3) in poultry [8,9,10,11], pork [12,13,14] and rabbit meat [15,16,17,18,19]. Providing increased amounts of n-3 essential fatty acids in human nutrition through the meat consumption can contribute to balance the n-6/n-3 PUFA ratio of the today’s consumer diet, thus preventing some correlated diseases such as hypercholesterolemia-related heart attack and strokes [4,7,20].

Fatty acids are involved in many technological aspects of meat quality. The main problem associated with the modification of the natural fatty acid profile of muscle foods is determined by the ability of unsaturated fatty acids, especially those with more than two double bounds, to oxidise and thus reduce the shelf-life of meat products [7]. This problem could also be more important when the meat with a high level of PUFA is used for further processing that involves mincing, long term frozen storage, and cooking [21]. Moreover, it has been widely reported that lipid oxidation represents a key-role in the development of cooked meat flavour. In swine, for example, it has been suggested that dietary use of linseed could impair the flavour of cooked meat when the α-linolenic (C18:3 n-3) acid content of the meat is above 3% of total fatty acids [14,22].

## 2. The Dietary Use of Linseed to Enhance n-3 PUFA Content of Rabbit Meat

Our research group carried out some studies in order to investigate the effect of different inclusion levels of whole linseed (up to 9%) in diets for growing rabbits on fatty acid composition, susceptibility to lipid oxidation, and sensory quality of the meat [23,24]. The experimental diets were formulated to meet the nutritional requirements of growing rabbits and linseed was added as a substitute for palm oil. Diets were offered to the rabbits during the last phase of growing period (3–4 weeks before slaughtering) and were supplemented with a supra-nutritional level of 200 mg/kg of feed α-tocopheryl acetate (vitamin E) in order to limit lipid oxidation in meat products [16].

### 2.1. Fatty Acid Composition

**Figure 1:**
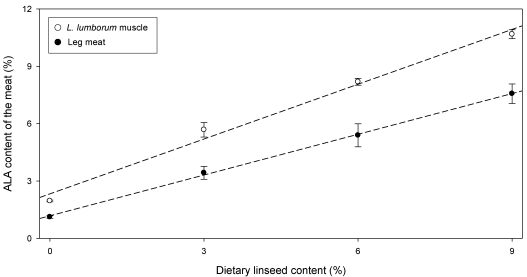
Relationship between dietary linseed and α-linolenic acid (ALA) content (mean ± sem) in rabbit meat (*L. lumborum* muscle: y = 0.71x + 1.18, R^2^ = 0.999; Leg meat: y = 0.96x + 2.33, R^2^ = 0.989) (n. of samples = 32) (from [24]).

The overall meat fatty acid composition was dramatically influenced by dietary linseed inclusion. With regard to the main categories of fatty acids, the linseed determined a lower content of total saturated fatty acids and a higher content of polyunsaturated fatty acids of *Longissimus lumborum* muscle and leg meat (data not shown) [23,24]. We also observed increasing levels of n-3 PUFA (P < 0.001) from control group (2.21% in *L. lumborum* and 2.39% in leg meat) towards groups fed 3% linseed (L3) (4.57% in *L. lumborum* and 5.96% in leg meat), 6% linseed (L6) (6.77% in *L. lumborum* and 8.48% in leg meat), and 9% linseed (L9) (8.85% in *L. lumborum* and 10.97% in leg meat) [24]. The increased content of n-3 PUFA was mainly due to the higher content of α-linolenic acid, which represents the main fatty acid of linseed. This result is shown in Figure 1, which reports the relationship between the content of whole linseed in the diet and the content of α-linolenic acid in rabbit meat. 

**Figure 2:**
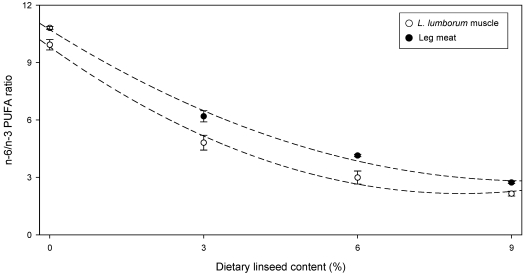
Relationship between dietary linseed content and n-6/n-3 PUFA ratio (mean ± sem) in rabbit meat (*L. lumborum* muscle: y = 0.09x^2^− 1.68x + 10.7, R^2^ = 0.995; Leg meat: y = 0.12x^2^− 1.91x + 9.81, R^2^ = 0.993) (n. of samples = 32) (from [24]).

The increased content of n-3 PUFA was able to reduce the n-6/n-3 PUFA ratio as evidenced in Figure 2. However the concentration of EPA and DHA found in both loin and leg meat was very low (about 0.1%) and not increasing from control to L9 group evidencing the limited efficiency of α-linolenic acid conversion to the long chain n-3 PUFA in rabbits. As stressed by Stanley *et al*. [25], n-6/n-3 PUFA ratio should not used alone as evaluation index of the nutritive value of meat. 

The effectiveness of whole linseed to increase the PUFA and α-linolenic acid contents of the meat has been previously reported by several studies on both rabbit [15,16,17,18,19] and other species [8,9,10,11,12,13,14,26]. It was also showed that administration of n-3 enriched diets during the last 3-4 weeks of the growing period is sufficient to achieve substantial fatty acid modification in loin and leg meat increasing the n-3 PUFA content to requested values, thus reducing the feed cost in comparison with a longer treatment. 

The recent scientific opinion from the European Food Safety Authority (EFSA) considered an intake for α-linolenic acid of 2 g/d consistent with recommended intakes for individuals in the general population in some European countries based on considerations of cardiovascular health [27]. Considering that 6% dietary linseed determined a n-3 PUFA content of 8.5% of the total fatty acids and a lipid content of 4.7 g/100g of leg meat, it can be estimated a content of 396 mg n-3 PUFA/100g meat which represents about 19% of recommended daily allowance (RDA) for n-3 PUFA by EFSA [27] (Table 1). 

**Table 1:** Influence of dietary linseed and type of muscle on total n-3 PUFA content of rabbit meat (mg/100g meat) and estimation of % recommended daily allowance (RDA) for n-3 PUFA based on a serving size of 100 g of meat.

Dietary linseed (%)	Loin ( *L. lumborum* muscle)		Leg	
	n-3 PUFA (mg/100g)	% RDA^1^	n-3 PUFA (mg/100g)	% RDA^1^
0	34	1.7	105	5.3
3	61	3.1	296	14.8
6	99	4.5	396	18.6
9	123	6.2	540	27.0

^1^ calculated on RDA of 2 g/d of n-3 PUFA as reported from EFSA [27].

The consumption of loin guarantees a lower intake of n-3 PUFA because of the lower total lipid content. This shows that enrichment of rabbit meat has the potential to provide a useful contribution intake of n-3 PUFA helping to balance n-6/n-3 PUFA ratio in rabbit meat products.

### 2.2. Susceptibility to Lipid Oxidation

**Figure 3:**
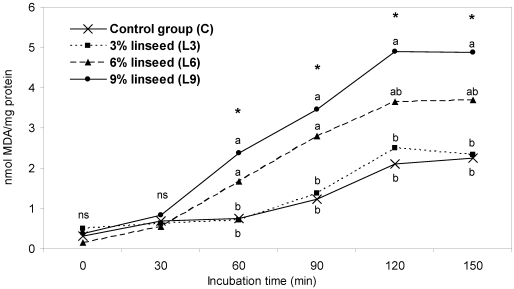
Influence of dietary linseed content on lipid susceptibility to oxidation (induced TBARS) of rabbit meat hamburgers (a, b = P<0.05) (n. of samples = 32) (modified from [24]).

With regard to the lipid susceptibility to oxidation, we did not observe any differences in *L. lumborum* muscle, whereas the leg meat exhibited a higher susceptibility of group fed 9% linseed (L9) in respect with the other groups, but only at the end of oxidation induction time (data not shown) [24]. Despite to these results, when a hamburger-type product was considered, the susceptibility to lipid oxidation was higher for L6 and L9 in comparison with L3 and control group which did not differ each other (Figure 3). Overall these results agree with the findings of an our previous study [23] which tested meat from rabbits fed on diets containing 0 or 8% linseed and indicate that lipid oxidation of the n-3 PUFA enriched meat becomes very critical especially when meat is further processed (i.e., mincing with high oxygen exposure of the meat).

### 2.3. Sensory Properties

Sensory analyses were carried out on hamburgers and indicated that the diet does not determine significant differences in the acceptability of the products produced with frozen meat batter stored for three or six months [24]. In the previous study [23], we observed no differences in the sensory characteristics of hamburgers produced with meat batters obtained from rabbits fed diets containing 0 or 8% linseed and frozen for three months of storage. However, at six months, differences in the sensory properties of the meat were detected.

## 3. Conclusions

Our studies on rabbit meat indicate that the dietary inclusion of linseed from 3 to 6% might be considered as a way both to achieve the enrichment of α-linolenic acid of the meat and guarantee a satisfactory product stability. This α-linolenic acid enrichment has the potential to provide a useful contribution intake of n-3 PUFA helping to balance n-6/n-3 PUFA ratio in rabbit meat products, even if it was confirmed a limited efficiency of its conversion to the long chain n-3 PUFA (EPA and DHA).

Higher levels of dietary linseed inclusion can be profitably used in products sold as whole carcass or cut-up which are less prone to undergo lipid oxidation compared to processed products. Particular attention should be given when meat with a high level of PUFA is used for further processing that involves mincing, cooking and long term frozen storage. Moreover, considering the overall results, the product quality concerning lipid oxidation and sensory properties can be considered quite good, despite the high level of unsaturation determined by the use of linseed. This might be also related to the positive effect exerted by α-tocopheryl-acetate (200 mg/kg feed).
